# Motivated semantic control: Exploring the effects of extrinsic reward and self‐reference on semantic retrieval in semantic aphasia

**DOI:** 10.1111/jnp.12272

**Published:** 2022-01-11

**Authors:** Nicholas E. Souter, Sara Stampacchia, Glyn Hallam, Hannah Thompson, Jonathan Smallwood, Elizabeth Jefferies

**Affiliations:** ^1^ Department of Psychology University of York UK; ^2^ Laboratory of Neuroimaging and Innovative Molecular Tracers (NIMTlab) Faculty of Medicine Geneva University Neurocenter University of Geneva Switzerland; ^3^ Department of Psychology University of Huddersfield UK; ^4^ School of Psychology and Clinical Language Sciences University of Reading UK; ^5^ Department of Psychology Queen’s University Kingston Ontario Canada

**Keywords:** Stroke, Semantic, Aphasia, Motivation, Reward, Self‐reference

## Abstract

Recent insights show that increased motivation can benefit executive control, but this effect has not been explored in relation to semantic cognition. Patients with deficits of controlled semantic retrieval in the context of semantic aphasia (SA) after stroke may benefit from this approach since ‘semantic control’ is considered an executive process. Deficits in this domain are partially distinct from the domain‐general deficits of cognitive control. We assessed the effect of both extrinsic and intrinsic motivation in healthy controls and SA patients. Experiment 1 manipulated extrinsic reward using high or low levels of points for correct responses during a semantic association task. Experiment 2 manipulated the intrinsic value of items using self‐reference, allocating pictures of items to the participant (‘self’) or researcher (‘other’) in a shopping game before participants retrieved their semantic associations. These experiments revealed that patients, but not controls, showed better performance when given an extrinsic reward, consistent with the view that increased external motivation may help ameliorate patients’ semantic control deficits. However, while self‐reference was associated with better episodic memory, there was no effect on semantic retrieval. We conclude that semantic control deficits can be reduced when extrinsic rewards are anticipated; this enhanced motivational state is expected to support proactive control, for example, through the maintenance of task representations. It may be possible to harness this modulatory impact of reward to combat the control demands of semantic tasks in SA patients.

## Background

Our ability to understand the world relies on flexible access to conceptual information within a semantic store (Jefferies, 2013). Evidence supports the existence of dissociable systems underlying the storage and retrieval of semantic representations (Lambon Ralph, Jefferies, Patterson, & Rogers, 2017). Semantic dementia patients with relatively focal atrophy focussed on the ventrolateral anterior temporal lobes show degraded semantic knowledge, while patients with semantic aphasia (SA) experience deregulated semantic retrieval, or semantic control, following left prefrontal and/or temporoparietal stroke (Jefferies & Lambon Ralph, 2006). Semantic control is an executive process which supports the retrieval of non‐dominant aspects of knowledge while overcoming competition from distractors (Hoffman, McClelland, & Lambon Ralph, 2018; Jefferies, 2013). Impaired semantic control in SA gives rise to deficits in both verbal communication and organisation of nonverbal actions (Jefferies, Thompson, Cornelissen, & Smallwood, 2019), consistent with the definition of SA as impaired manipulation of verbal and non‐verbal symbolic information (Head, 1926). In line with the damage to the left ventrolateral prefrontal cortex and/or left temporoparietal regions in SA, studies of healthy participants using neuroimaging (Jackson, 2021) and transcranial magnetic stimulation (Hallam, Whitney, Hymers, Gouws, & Jefferies, 2016) have implicated both the left inferior frontal gyrus (LIFG) and posterior middle temporal gyrus (pMTG) in semantic control.

In SA, access to semantic knowledge is not universally compromised, but depends on task demands. Semantic retrieval is impaired for subordinate meanings and when inhibition of task‐irrelevant distractors is required (Jefferies, 2013). This results in reduced flexibility when retrieving semantic information in ambiguous contexts (Noonan, Jefferies, Corbett, & Lambon Ralph, 2010). Impaired semantic control in SA is also evident when retrieving thematic associations between concepts: identifying weak as opposed to strong associations requires semantic control processes that focus retrieval on non‐dominant conceptual information (Thompson et al., 2017). Research has explored manipulations which ameliorate semantic control deficits in SA, such as cueing. Successive phonemic cues (e.g., c.., ca.., and cam. for camel) can facilitate picture naming (Jefferies, Patterson, & Lambon Ralph, 2008), while contextually relevant sentences (Noonan et al., 2010) or emotional and location cues (Lanzoni et al., 2019) can facilitate the retrieval of non‐dominant interpretations of ambiguous homonyms. Cues reduce control demands by narrowing down the number of retrievable options and biasing retrieval toward task‐relevant information.

An alternative approach to facilitating semantic retrieval involves recruiting processes beyond semantic cognition. Investigations with healthy adults have demonstrated that extrinsic rewards, such as monetary incentives or awarded points, can improve performance in domains including control of visual attention (Padmala & Pessoa, 2011), task‐switching (Capa, Bouquet, Dreher, & Dufor, 2013), contextual control (Kouneiher, Charron, & Koechlin, 2009), creative problem solving (Cristofori, Salvi, Beeman, & Grafman, 2018), interference control (Zhao, Jia, & Maes, 2018), and conflict adaptation (Dreisbach & Fischer, 2012). Behavioral benefits of extrinsic reward include increased accuracy, reduced reaction times, and reduced switch‐costs (Yee & Braver, 2018). Extrinsic incentives are considered a key element of ‘gamification’ (Mekler, Brühlmann, Tuch, & Opwis, 2017), which uses typical elements of digital games to increase engagement with training activities, including post‐stroke rehabilitation (Romani, Thomas, Olson, & Lander, 2019). To our knowledge, extrinsic rewards have not been used previously in conjunction with semantic tasks or in SA patients.

Tasks with high control demands are effortful as they draw on limited resources including selective attention and working memory (Yee & Braver, 2018). The cost of mental exertion is typically weighed against the potential benefits of the action (Botvinick & Braver, 2015). As such, tasks perceived as high effort and low in reward may be less appealing than more trivial low‐effort and high‐reward actions. Introducing task‐based incentives can offset perceived costs (Goschke & Bolte, 2014) and increase preparatory control, and therefore one’s ability to sustainably engage with a task (Notebaert & Braem, 2015). This can benefit either cognitive stability or flexibility, depending on recent reward history (Fröber, Pfister, & Dreisbach, 2019). The neural processing of extrinsic reward has been consistently linked to a network of regions, including the ventromedial prefrontal cortex, caudate, and putamen (Lin, Adolphs, & Rangel, 2012). Cumulative reward value appears to be tracked and represented in these regions (Juechems, Balaguer, Ruz, & Summerfield, 2017). Effects of reward on cognition have been attributed to dopaminergic transmission between these regions and the multiple demand network (MDN), which supports challenging tasks across domains (Camilleri et al., 2018; Parro, Dixon, & Christoff, 2018).

While extrinsic reward refers to incentives provided externally, intrinsic reward refers to inherent enjoyment of or interest in a task (Mori et al., 2018). Intrinsic motivation is relatively difficult to manipulate experimentally, but can be modulated indirectly, through factors such as self‐reference. Tamir and Mitchell (2012) demonstrated that self‐referential information is intrinsically motivating; participants reliably choose to forgo monetary incentives in order to disclose information about the self, in conjunction with increased activation in brain regions associated with reward processing. The neural substrates underlying both intrinsic motivation and self‐reference show considerable overlap with reward circuitry (Di Domenico & Ryan, 2017; Enzi, de Greck, Prösch, Tempelmann, & Northoff, 2009). Cognition shows biases in favor of self‐referenced items within perception (Sui, He, & Humphreys, 2012), attention (Sui & Humphreys, 2015b), working memory (Röer, Bell, & Buchner, 2013), and recognition memory (Hou, Grilli, & Glisky, 2019). Moreover, Sui and Humphreys (2015a) demonstrated that extrinsic reward and self‐reference confer separable but equivalent benefits in associative learning. Self‐reference benefits to episodic memory persist in patients with SA (Stampacchia et al., 2019).

If regions associated with reward processing are intact, it may be possible to harness modulatory effects of motivation in rehabilitation for post‐stroke aphasia. The benefits of increased motivation may be more pronounced in more impaired patients, with greater difficulties constraining internal representations increasing reliance on external prompts. Given the evidence of effects of reward and self‐reference on cognitive functions across domains, similar benefits may occur for semantic control. However, semantic control is dissociable from domain‐general control: the peak activations in fMRI studies that manipulate semantic control demands in healthy participants fall outside the multiple‐demand network (Gao et al., 2021; Noonan, Jefferies, Visser, & Lambon Ralph, 2013), and inhibitory stimulation of semantic control sites temporarily disrupts control‐demanding semantic tasks, but not demanding visual judgements (Whitney, Kirk, O’Sullivan, Lambon Ralph, & Jefferies, 2011). Moreover, while impaired semantic control is ubiquitous in SA, some but not all of these patients have general deficits of cognitive control: lesion‐symptom mapping shows that these semantic and non‐semantic control deficits are associated with different patterns of structural damage (Souter, Wang, et al., 2021). Given this distinction, there is a need to investigate the effects of motivation in tasks with high semantic control demands to establish if this domain can benefit from ‘gamification’ strategies to the same degree as other cognitive tasks. Furthermore, evidence suggests affective abnormalities in SA, including the ability to categorise facial portrayals according to discrete emotion categories (Souter, Lindquist, & Jefferies, 2021). This is thought to reflect deficits in constraining internal states beyond the conceptual domain, which may extend to and, therefore, limit modulatory effects of motivation. While people with aphasia generally benefit from the use of motivating ‘gamification’ strategies, we cannot assume that these benefits will transfer to people with SA for this reason. This is a key motivation for the current study. SA patients have been shown to benefit from the provision of external cues that provide additional information pertaining to semantic decisions (Noonan et al., 2010), but the influence of reward manipulations, which provide external prompts in the absence of contextually‐relevant information, has not been investigated to our knowledge.

The current study aimed to investigate the influence of both extrinsic reward and intrinsic motivation induced through self‐reference on the retrieval of strong and weak thematic associations in SA. Experiment 1 assessed the effect of cued extrinsic reward in the form of high or low levels of performance‐contingent token points. Experiment 2 assessed the effect of self‐reference by allocating pictured ‘shopping items’ either to the participant (‘self’ condition) or the researcher (‘other’ condition), prior to semantic judgements about these items. If modulatory effects of motivation can benefit semantic control, high extrinsic reward and/or self‐reference might ameliorate patients’ semantic deficits. Motivation may support the maintenance of task goals when semantic control is deficient; consequently, any performance gains would likely be greater for weak associations, which place higher demands on semantic control. A better understanding of the effects of motivation on semantic retrieval of strong and weak associations may have implications for the use of gamified approaches for aphasia rehabilitation.

## Method

### Participants

The sample consisted of 16 SA patients (nine females) and 15 controls (12 females). All participated in Experiment 1, while a subset participated in Experiment 2 (demographic information is presented separately in the ‘Participants’ sections within each experiment). Patients were recruited from communication support groups across Yorkshire. All had aphasia following left hemisphere stroke and were at least 18 months post stroke. Patients were selected to show impairments in both verbal and non‐verbal semantic cognition, consistent with previous definitions of SA (Jefferies & Lambon Ralph, 2006). The criteria used are explained in the Supplementary Section Background Neuropsychology. Controls were healthy adults matched to the patients on age and years in education and reported no history of psychiatric or neurological disorders. Informed consent was obtained for all participants.

### Lesion analyses

**Figure 1:**
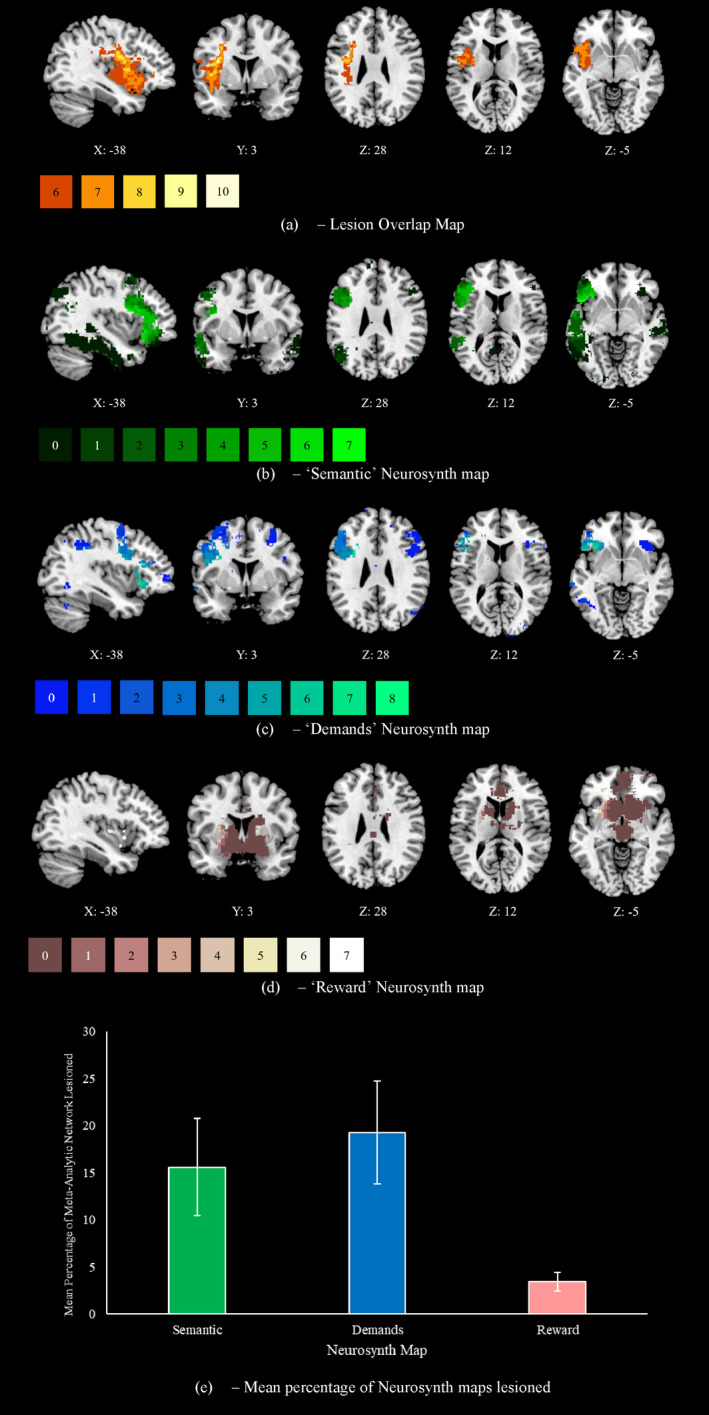
Patient lesion analyses, including (a) a lesion overlap map for ten SA patients in the current study, created using manual segmentation in MRICron. This map shows lesion overlap in six or more patients, with the color of the lesioned area corresponding to the number of affected cases (bottom left). We assessed the extent of overlap between patient lesions and term‐based meta‐analytic maps from Neurosynth for the terms (b) ‘semantic’ (1031 studies), (c) ‘demands’ (596 studies), and (d) ‘reward’ (922 studies). Neurosynth maps are colored according to impact by lesions across the sample, with brighter areas reflecting those more often implicated in lesions. Each map was formatted in MRICron. We present (e) the mean percentage of each map lesioned across patients, with standard error of the mean error bars.

Ten patients (P1–P10) had MRI scans at the York Neuroimaging Centre (see Supplementary Section MRI Acquisition for scanning protocols and extraction/registration procedure). All ten participated in Experiment 1 and four (P3–P6) participated in Experiment 2. Each patient’s lesion was manually traced in MRIcron. Figure 1a provides a lesion overlap map for these patients. Eight cases showed damage to the LIFG. Several patients showed damage to other regions, including the pMTG, superior temporal gyrus, and supramarginal gyrus. Clinical acute‐stage scans were available for two further patients and revealed damage to the LIFG (MRI for P16) and a left frontoparietal lesion (CT for P12). Lesion information was not available for the remaining four patients due to contraindications for scanning and/or closure of scanning facilities during the COVID‐19 pandemic.

To assess the impact of patients’ lesions on functional networks of interest, we extracted maps from Neurosynth using term‐based meta‐analyses (Yarkoni, Poldrack, Nichols, Van Essen, & Wager, 2011) for ‘Semantic’, ‘Demands’, and ‘Reward’ (Figure 1b–d). This allowed us to observe the extent to which patients present with damage to regions associated with semantic processing, domain‐general task demands, and reward processing. We calculated the average percentage of each map that was damaged across the patients with available lesion maps. Analysis was restricted to left hemisphere aspects of each network, such that it reflects a percentage of all voxels that could possibly be lesioned in an exclusively left‐hemisphere stroke sample. While right‐hemisphere aspects of these networks may be affected by disconnection (Souter, Wang, et al., 2021), this is beyond the scope of the current article. As seen in Figure 1e, patients showed the most damage to ‘Demands’ regions followed by ‘Semantic’ regions. ‘Reward’ regions were relatively spared, suggesting that it might be possible to harness modulatory impacts of reward.

### Background neuropsychological testing

Patients completed background tests of language, memory, executive function, and semantic cognition. The control participants tested on the experimental tasks in this study did not complete these background assessments. Patients’ individual scores are provided in Table 1 for background neuropsychology and Table 2 for semantic tests. Interpretation of the sample’s performance is provided in the Supplementary Background Neuropsychology section. Patients presented with variable levels of impairment in speech fluency and word repetition. Most patients presented with impaired working memory. Visuospatial processing was largely preserved. Eleven patients showed impairment on at least one test of executive function

**Table 1:** Patient performance on background neuropsychological testing

Test	Max	Cut‐off	Patients mean	P1	P2	P3	P4	P5	P6	P7	P8	P9	P10	P11	P12	P13	P14	P15	P16	# Impaired
*Non‐semantic language tests*																				
PALPA 9 real‐word repetition	80	73^a^	60.93	NA	**71**	**42**	**7**	78	79	**1**	NA	74	77	79	75	**67**	**50**	74	79	6
Cookie theft (words/min)	–	50^b^	36.79	**0**	**18**	**9**	**38**	60	**37**	**0**	NA	54	**37**	77	80	**34**	NA	**16**	55	9
Category Fluency (eight categories) (words/min)	–	7.75	5.19	NA	**3.3**	**1.9**	8.6	**1.8**	**3.3**	NA	NA	10	**7.1**	9.4	11.3	**3.8** ^c^	**0** ^c^	**3.8**	**3.5**	9
Letter Fluency (F, A, S) (words/min)	–	7.27	2.54	NA	**0.7**	**0.7**	**4**	**1**	**2**	NA	NA	**5.3**	**3**	**4.3**	**4.3**	**1**	**1.3**	**2.7**	**2.7**	13
*Verbal working memory*																				
Digit span forward	8	5.54	3.00	**0**	**4**	**1**	6	6	**4**	NT	**0**	**4**	**4**	**4**	**4**	**3**	**0**	**2**	**3**	13
Digit span backward	7	3.66	1.27	**0**	**2**	**0**	4	**2**	**0**	NT	**0**	**2**	**3**	**2**	**2**	**0**	**0**	**0**	**2**	14
*Visuospatial processing*																				
VOSP dot counting	10	8	9.25	**7**	10	10	10	10	10	8	8	10	10	10	9	9	9	8	10	1
VOSP position discrimination	20	18	18.87	19	20	**15**	20	**17**	20	19	20	20	20	20	20	**17**	20	**15**	20	4
VOSP number location	10	7	8.44	8	10	**5**	8	10	10	10	8	**5**	10	10	10	**4**	9	8	10	3
VOSP cube analysis	10	6	8.25	8	9	**4**	8	7	9	10	9	10	10	9	10	**5**	9	**5**	10	3
*Executive and spatial processing*																				
TEA: counting without distraction	7	4.2	5.53	**2**	5	6	5	**4**	NT	7	5	5	7	5	6	6	7	6	7	2
TEA: counting with distraction	10	2.6	3.73	**1**	3	**1**	3	**2**	NT	7	**1**	**2**	6	**2**	9	4	**2**	3	10	7
Raven's colored matrices	36	28^a^	29.00	31	**27**	31	33	**19**	30	34	**24**	**21**	33	34	32	**20**	32	**27**	36	6
Brixton spatial anticipation	54	28	26.94	**21**	**7**	**18**	39	**24**	**23**	31	34	31	30	41	32	30	**21**	**18**	31	7
Trail Making Test A	24	24^a^	22.88	**19**	**22**	**23**	24	24	**23**	24	24	24	24	24	24	24	**16**	**23**	24	6
Trail Making Test B	23	17.4^a^	17.37	**2**	23	**16**	21	**1**	**5**	23	23	19	22	22	20	19	20	20	22	4

Note

Scores are number of correct responses unless otherwise specified. NT = unavailable for testing; NA = testing was not attempted because patients were non‐fluent; TEA = Test of Everyday Attention, elevator counting subtest (Robertson, Ward, Ridgeway, & Nimmo‐Smith, 1994); VOSP = Visual Object and Space Processing battery (Warrington & James, 1991). Cutoffs for impairment correspond to two standard deviations below control mean performance (separate from the control sample used in the current study), with impaired scores underlined and in bold. These are taken from control norms from respective test manuals, unless otherwise specified (see below).

^a^ Cutoffs taken from control testing at the University of York. Number of controls: Raven’s (Raven, 1962)= 20; Trail Making Test (Reitan, 1958)= 14

^b^ Cutoff for cookie theft description (Goodglass, Kaplan, & Barresi, 2001) reflects the ‘very slow’ classification taken from Kerschensteiner, Poeck, and Brunner (1972)

^c^ Patients P13 and P14 completed only four categories (animals, fruit, birds, and breeds of dog) for the test of category fluency, while the other patients completed eight (animals, fruit, birds, breeds of dog, household objects, tools, vehicles, and types of boat).

**Table 2:** Patient performance on the Cambridge Semantic Battery and tests of semantic control

Test	Max	Cutoff	Patient mean	P1	P2	P3	P4	P5	P6	P7	P8	P9	P10	P11	P12	P13	P14	P15	P16	# Impaired
Semantic composite score	–	–	–	‐1.36	‐1.21	‐1.07	1.32	.37	.34	.63	.40	.63	.96	1.26	.92	‐.75	‐1.69	‐.89	.13	–
*Cambridge semantic battery*																				
Picture Naming (no cues)	64	59	41.00	**1**	61	**19**	**46**	60	**50**	**3**	**0**	**56**	62	59	63	**54**	**33**	**51**	**38**	11
Picture Naming (with cues)	64	–	51.19	3	63	58	64	64	64	10	0	62	64	64	64	63	52	63	61	–
Word–Picture Matching	64	62.7	60.88	63	**62**	**60**	63	**62**	**62**	**52**	**56**	64	**62**	64	**62**	**62**	**58**	**59**	63	11
Word CCT	64	56.6	49.88	**39**	**43**	**29**	**56**	59	**52**	57	**56**	61	60	59	59	**48**	**28**	**45**	**47**	9
Picture CCT	64	52.7	50.94	**31**	**44**	**45**	61	**45**	57	54	61	53	61	62	59	**44**	**35**	**51**	**52**	8
*Ambiguity task*																				
Miscued dominant	30	30	18.93	**12**	**13**	**13**	**26**	**20**	**19**	**21**	NT	**24**	**26**	**22**	**25**	**16**	**12**	**21**	**14**	15
Miscued subordinate	30	26.6	15.20	**7**	**10**	**14**	28	**10**	**15**	**18**	NT	**18**	**19**	**22**	**18**	**13**	**12**	**13**	**11**	14
No cue dominant	30	28.4	24.38	**22**	**18**	**24**	**27**	**24**	**26**	**27**	**25**	**28**	**28**	**27**	30	**25**	**11**	**22**	**26**	15
No cue subordinate	30	27.6	16.81	**11**	**9**	**14**	**21**	**19**	**17**	**19**	**16**	**21**	**19**	28	**26**	**12**	**14**	**12**	**11**	15
Cued dominant	30	30	24.13	**23**	**21**	**19**	**29**	**24**	**23**	**23**	NT	**27**	**29**	**28**	30	**23**	**19**	**24**	**20**	14
Cued subordinate	30	28.8	21.87	**25**	**14**	**20**	28	**19**	28	**24**	NT	**23**	**25**	**26**	29	**19**	**16**	**15**	**17**	12
*Synonym with distractors*																				
Strong distractors	42	35.4	19.94	**15**	**12**	**13**	38	**21**	**23**	**30**	**16**	**22**	**17**	**27**	**13**	**17**	**17**	**13**	**25**	15
Weak distractors	42	40.4	29.31	**25**	**23**	**29**	**36**	**27**	**30**	**31**	**33**	**28**	**39**	**35**	**35**	**25**	**16**	**24**	**33**	16
*Object use*																				
Alternative	37	33.67	22.06	**14**	**13**	**14**	**32**	34	**22**	**22**	**22**	**26**	**29**	**31**	**29**	**12**	**17**	**9**	**27**	15
Canonical	37	35.9	33.56	**32**	**31**	**29**	37	37	**35**	**33**	**35**	37	37	**34**	**35**	**29**	**34**	**28**	**34**	12

Note

Scores are number of correct responses. NT = unavailable for testing, CCT = Camel and Cactus Test. Cutoffs for impairment are taken from testing at the University of York and correspond to two standard deviations below mean control performance (separate from the control sample used in the current study), with impaired scores underlined and in bold. Number of controls: Cambridge Semantic Battery (Bozeat, Lambon Ralph, Patterson, Garrard, & Hodges, 2000)= 10, Ambiguity task (Noonan et al., 2010), Synonym with distractors (Samson, Connolly, & Humphreys, 2007), Object use (Corbett, Jefferies, & Lambon Ralph, 2011)= 8. Semantic composite score reflects regression scores derived from principal component analysis, including tests with high control demands [CTT words, CCT pictures, Ambiguity task (no cue: dominant + subordinate), Object use task (alternative + canonical), and Synonym with distractors (strong + weak)]. Lower composite scores reflect greater impairment.

All patients were impaired on at least one verbal and one non‐verbal semantic task. All patients performed close to ceiling level on word‐picture matching, reflecting low control demands. On word and picture versions of the Camel and Cactus Test of semantic association, half of the sample showed impairment. Patients presented with considerable variation in picture naming, although performance was improved by successive phonemic cues in all who were able to name at least one picture. Patients presented with the anticipated impairment in tests manipulating semantic control demands, including difficulty retrieving subordinate conceptual information, susceptibility to cues and miscues, and difficulty rejecting strong thematic distractors.

Principal component analysis of the semantic tasks using oblique rotation revealed two components with Eigenvalues greater than 1 (Table 3). The first component reflected performance on tasks with high semantic control demands: these factor scores were used as a semantic control composite for each participant. Lower scores reflect greater impairment. This semantic factor was positively correlated with performance on the Brixton Spatial Anticipation Test (Burgess & Shallice, 1997): *r*_s_(14) = .837, *p* < .001. It did not relate to performance on any other manipulation of executive function (*p* ≥ .200, see Supplementary Section Background Neuropsychology). The second semantic factor loaded on tasks involving object identification.

**Table 3:** Pattern matrix for principal component analysis of SA patients’ performance on semantic tests with oblique rotation

Task	Component 1 (Eigenvalue = 4.03)	Component 2 (Eigenvalue = 1.52)
CCT words	.**876**	.083
CCT pictures	.**896**	−.078
Picture naming	.089	.**877**
Word–picture matching	−.062	.**916**
Ambiguity task	.**900**	.057
Synonym judgement task	.**903**	−.154
Object use task	.**801**	.156

Note

Strong loadings for each component are in bold. CCT = Camel and Cactus Test.

## Experiment 1: The effect of cued extrinsic reward on semantic retrieval

### Method

#### Participants

This sample included 16 patients (nine females) with a mean age of 64.4 years (*SD* = 12.3), a mean age of leaving education of 17.5 years (*SD* = 2.9), and a mean of 11.3 years (*SD* = 6.6) since stroke. These patients were compared with 15 controls (12 females) with a mean age of 70.7 (*SD* = 9.7) and a mean age of leaving education of 18.8 years (*SD* = 3.9). Patients and controls were matched for age [*U* = 74.0, *p* = .069] and age of leaving education [*U* = 102.0, *p* = .469].

#### Design

This experiment used a repeated measured design, with all participants making strong and weak thematic associations under the conditions of high and low reward. A three‐alternative forced choice format was used: participants were asked to select a target word, presented alongside two foils, based on the strongest thematic association to a probe word. The experiment was conducted over two sessions, each consisting of four high and four low reward blocks. Each block contained eight trials split equally across strong and weak associations. High and low reward blocks were alternated. There were 64 trials per session and 128 trials in total. There was no difference across sessions for accuracy or response time: *p* ≥ .190.

#### Stimulus properties

Descriptive statistics for Experiment 1 stimulus properties are reported in Table S1. Target and probe words were taken from the Edinburgh Associative Thesaurus, a publicly available dataset of associative strength between words (Kiss, Armstrong, Milroy, & Piper, 1973). Probes and targets were more related in strong than weak association trials: *t*(67.6) = 42.1, *p* < .001. There were no differences in association strength across blocks [*t* < 1] or sessions [*t* < 1].

We examined frequency, imageability, and length of the target and probe words. Subtlex‐UK (Van Heuven, Mandera, Keuleers, & Brysbaert, 2014) was used to obtain word frequency. Sources for imageability ratings included the MRC Psycholinguistic Database (Coltheart, 1981), N‐Watch (Davis, 2005), The Glasgow Norms (Scott, Keitel, Becirspahic, Yao, & Sereno, 2019), Bird, Franklin, and Howard (2001a; 2001b), Cortese and Fugett (2004a, 2004b), and Davey et al. (2015). Frequency and imageability scores were obtained on 7‐point Likert scales and were averaged when multiple sources were available. Overall, 17% of frequency scores could not be retrieved. Missing scores were largely for compound words such as ‘snooker ball’ or ‘space suit’. Imageability ratings were obtained for all but one word. For both target and probe words, three separate 2 × 2 ANOVAs were run with frequency, imageability, and length as dependent variables, examining effects of reward (high/low) and association strength (strong/weak). These ANOVAs are reported in Table S3. All psycholinguistic properties were matched across reward condition and association strength [*p* ≥ .071].

#### Procedure

**Figure 2:**
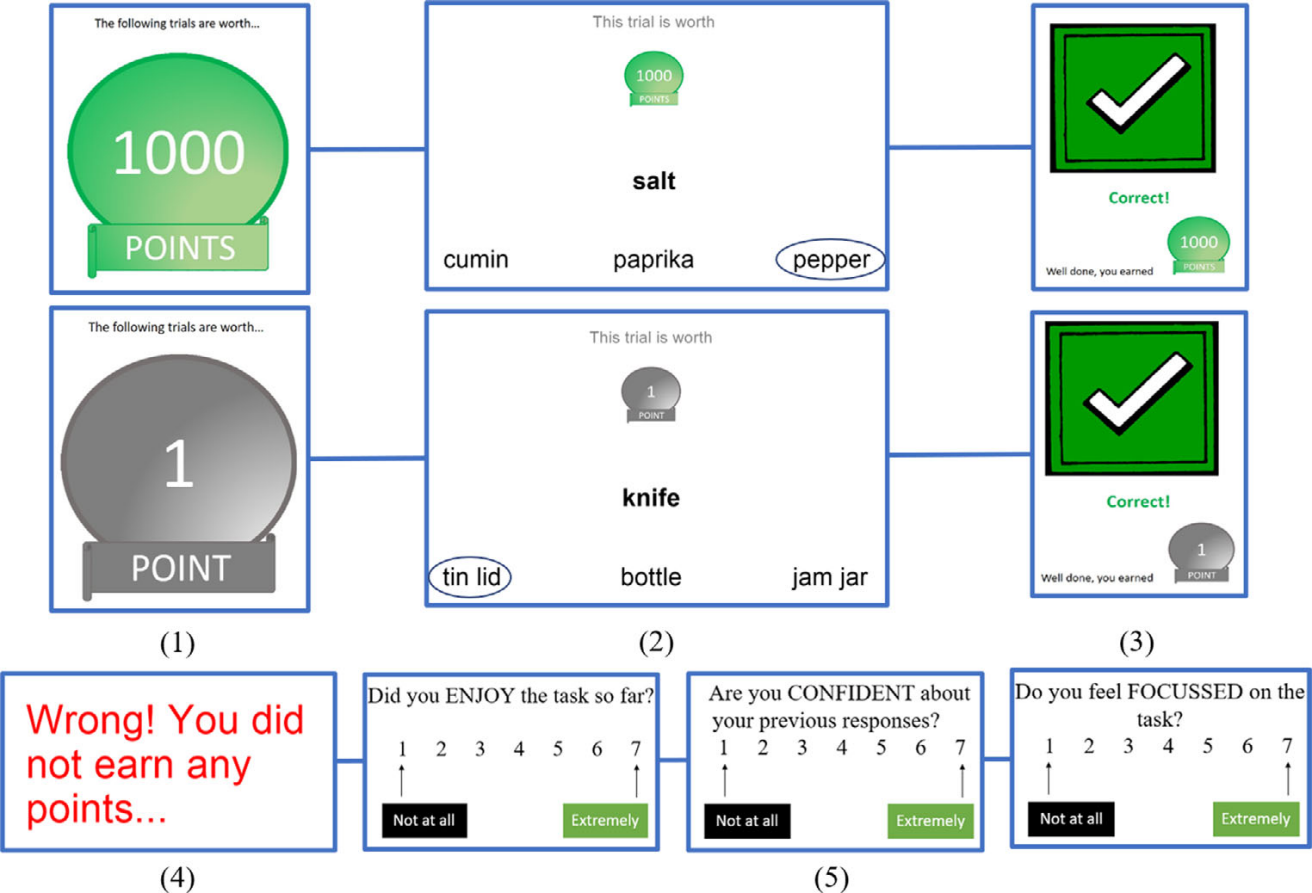
Experiment 1 procedure. (1) Each block was preceded by a high reward or low reward graphic. (2) Participants made thematic associations, either with strong or weak associations. Participants were provided with feedback as to whether their response was (3) correct or (4) incorrect. (5) Following each block, participants completed ratings of enjoyment, confidence, and focus.

Each session was preceded by an instruction phase, which included two practice trials to familiarise participants with the procedure. Stimuli for the main experiment were presented on a laptop using PsychoPy3 (Peirce et al., 2019). Each block was preceded by a graphic, informing the participant that correct answers were each worth 1 point (low reward) or 1,000 points (high reward). Due to impaired reading ability, the researcher read all words aloud to the patients. Patients indicated their response by pointing to the screen, with the researcher pressing the corresponding key. Control participants read the words themselves and keyed in their own responses. Responses were followed by feedback, informing the participant that they had won either 1 or 1,000 points or that they were incorrect and had not won any points. If a response was not given within ten seconds, participants were informed that they had not won any points. The prospect of gaining points was abstract and not linked to monetary gain. Each block was followed by self‐report ratings of task enjoyment, response confidence, and task focus, each on a 7‐point Likert scale. Figure 2 provides a summary of the procedure for Experiment 1.

#### Data analysis

Accuracy (proportion of correct responses) was our key dependent measure. As we were specifically interested in effects of reward on patients’ accuracy for weak associations, we first ran a repeated measures ANOVA for the patients alone, observing the effects of reward (high/low) and association strength (strong/weak) as within‐subject independent variables. Accuracy was then entered into an omnibus‐mixed ANOVA, adding group (patients/controls) as a between‐subjects variable. Post hoc contrasts for significant interactions are reported with Bonferroni‐correction applied. Mixed ANOVAs were conducted for ratings of enjoyment, confidence, and focus, examining effects of reward and group. Analysis and interpretation of participants’ response time can be seen in Table S4.

### Results

**Figure 3:**
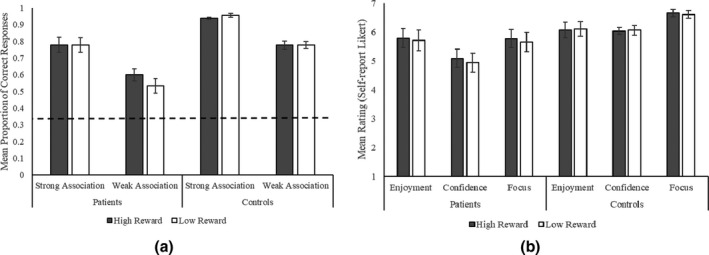
Experiment 1 bar graphs for (a) mean proportion of correct response (dotted line reflects chance performance, 0.33) and (b) self‐report ratings across reward conditions, participant groups, and association strength, with standard error of the mean error bars.

Figure 3 shows participants’ mean accuracy and self‐report ratings across reward condition, group, and association strength. Table S5 provides descriptive statistics for Experiment 1.

#### Effects of reward on semantic retrieval in patients

The patient group ANOVA revealed significant main effects of strength [*F*(1, 15) = 147.7, *p* < .001, $\eta_{p}^{2}$ = .91] and reward [*F*(1, 15) = 5.4, *p* = .034, $\eta_{p}^{2}$ = .27] and a significant reward by strength interaction [*F*(1, 15) = 7.0, *p* = .019, $\eta_{p}^{2}$ = .32]. Patients had higher accuracy on high than low reward trials and on strong than weak association trials. Post hoc contrasts for the interaction demonstrated that patients were more accurate for weak associations in the high than low reward condition [*t*(15) = 3.3, corrected *p* = .010]. There was no effect of reward on strong associations [*t* < 1]^1^. Patients’ semantic control composite scores positively correlated with their overall accuracy [*r*_s_(14) = .70, *p* = .003], reflecting higher accuracy in less‐impaired patients. There was no association between the semantic composite and the effect of reward [strong: *r*_s_(14) = .16, *p* = .563, weak: *r*_s_(14) = −.08, *p* = .780].

#### Omnibus ANOVA comparing effects across patients and controls

ANOVA results are shown in Table 4. Controls were more accurate than patients overall. Accuracy was higher for strong than weak association trials. There was a reward by group interaction, with a larger difference in accuracy between high and low reward trials for patients [*t*(15) = 2.3, corrected *p* = .068] than controls [*t* < 1],^2^ although neither contrast survived correction. There was a reward by strength interaction, with a greater difference in accuracy between the high and low reward conditions for weak [*t*(30) = 2.1, corrected *p* = .096] than for strong association trials [*t* < 1]^3^, although again neither contrast survived correction. The three‐way reward by strength by group interaction was not significant.

**Table 4:** Omnibus ANOVA results for all Experiment 1 (extrinsic reward) dependent variables

Dependent variable	Main effect/interaction	Results
Accuracy	Group	*F*(1, 29) = 18.9, *p* < .001, $\eta_{p}^{2}$ = .40^a^
	Reward	*F*(1, 29) = 1.3, *p* = .263, $\eta_{p}^{2}$ = .04
	Reward by group	*F*(1, 29) = 4.8, *p* = .037, $\eta_{p}^{2}$ = .14^a^
	Strength	*F*(1, 29) = 215.3, *p* < .001, $\eta_{p}^{2}$ = .88^a^
	Strength by group	*F*(1, 29) = 2.9, *p* = .100, $\eta_{p}^{2}$ = .09
	Reward by strength	*F*(1, 29) = 5.6, *p* = .025, $\eta_{p}^{2}$ = .06^a^
	Reward by strength by group	*F*(1, 29) = 2.0, *p* = .169, $\eta_{p}^{2}$ = .06
Enjoyment	Group	*F*(1, 29) = .6, *p* = .438, $\eta_{p}^{2}$ = .02
	Reward	*F*(1, 29) = .2, *p* = .690, $\eta_{p}^{2}$ < .01
	Reward by group	*F*(1, 29) = .6, *p* = .441, $\eta_{p}^{2}$ = .02
Confidence	Group	*F*(1, 29) = 7.7, *p* = .010, $\eta_{p}^{2}$ = .21^a^
	Reward	*F*(1, 29) = 1.4, *p* = .244, $\eta_{p}^{2}$ = .05
	Reward by group	*F*(1, 29) = 2.7, *p* = .112, $\eta_{p}^{2}$ = .09
Focus	Group	*F*(1, 29) = 6.6, *p* = .016, $\eta_{p}^{2}$ = .19^a^
	Reward	*F*(1, 29) = 5.8, *p* = .023, $\eta_{p}^{2}$ = .17^a^
	Reward by group	*F*(1, 29) = .8, *p* = .379, $\eta_{p}^{2}$ = .03

^a^ Reflects a significant result at the .05 level.

Ratings of enjoyment and confidence were not influenced by reward. Controls reported significantly higher confidence and focus than patients. A main effect of reward was found for focus, with higher ratings in the high than low reward condition. As all ratings were taken at the block level, it was not possible to investigate effects of association strength. There were too few participants in the current sample to assess the relationship between these ratings and accuracy.

### Experiment 1 summary

Experiment 1 studied effects of cued extrinsic reward on SA patients’ and controls’ ability to retrieve thematic associations. An ANOVA for the patient group demonstrated that high reward improved accuracy for weak but not strong associations, suggesting that high extrinsic reward can aid the retrieval of semantic associations when semantic control is deficient. Results from the omnibus ANOVA suggest that benefits of extrinsic reward were greater for the patients than for controls, and for weak than strong associations. Self‐reported focus was also higher in the high than low reward condition.

## Experiment 2: The effect of self‐reference on semantic retrieval

### Method

#### Participants

Experiment 2 included a subset of Experiment 1 participants. This included ten SA patients (six females) with a mean age of 62.4 years (*SD* = 10.1), a mean age of leaving education of 18.3 years (*SD* = 3.4), and a mean of 10.1 years (*SD* = 5.4) since stroke. Eleven control participants (eight females) were included in this sample with a mean age of 69.9 (*SD* = 10.3) and a mean age of leaving education of 18.9 (*SD* = 3.6). There was no significant difference between groups for age [*U* = 29.0, *p* = .067] or age leaving education [*U* = 52.0, *p* = .831].

#### Design

This experiment used a repeated‐measures design, with participants making strong and weak thematic associations across ‘self’ and ‘other’ conditions. A three‐alternative forced choice format was used. Probe pictures were used as these were viewed as fitting in the context of the shopping game used to reinforce self‐referential encoding (explained in ‘Procedure’ section). Pictures were selected for 28 pairs of semantically related items (e.g., harp‐lute, ant‐wasp). For each pair, one picture was allocated to the participant (‘self’) and one to the researcher (‘other’), counterbalanced across participants. Self and other trials were presented in a random order. The experiment was conducted over two sessions, each containing 56 trials. During the first session, participants completed strong and weak associations for one item in each pair. During the second session, participants completed the same associations for the remaining probes. Foils were thematically related to the target and were also kept consistent across both objects in each pair. Session order was counterbalanced. There was no difference across sessions for accuracy or response time: *p* ≥ .259.

#### Stimulus properties

Descriptive statistics for Experiment 2 stimulus properties are reported in Table S2. Association strength between the probe pictures and target words was validated using ratings from an independent sample of healthy adults on a 7‐point Likert scale. Ratings were collected over three surveys, with sample size ranging between 30 and 42. For both probes within each pair, equivalent strong and weak associations were generated. For example, pictures of a harp and a lute were equally strongly and weakly associated with the target words strings and vinyl, respectively. Associations between probes and targets were rated as stronger on strong than on weak association trials: *p* < .001. Association strength within strong and weak association categories was matched across self‐reference conditions and sessions: *p* ≥ .793.

Frequency, imageability, and length were examined for the target words (using the same sources detailed in Experiment 1 ‘Stimulus properties’ section). Ratings of frequency and imageability could not be retrieved for 14% and 7% of the target words, respectively. This was largely the case for compound words. One‐way ANOVAs were run for each factor, looking for effects of association strength (see Table S3). No effects of association strength were found [*p* ≥ .195]. Due to counterbalancing, it was not necessary to compare psycholinguistic ratings across conditions or sessions.

#### Procedure

At the start of both sessions, participants completed the allocation phase ‘shopping game’, intended to reinforce self‐referential encoding. Both the participant and researcher had a ‘shopping list’ in front of them, respectively labeled ‘My shopping list’ and ‘*researcher’s name*’s shopping list’, including pictures and names of each object allocated to them. The researcher and participant took turns finding the items on their lists and placing them into their respective baskets. Participants searched through a pile of laminated pictures, found the next item on their list, and placed it into their basket. Throughout this process, the researcher provided verbal prompts to reinforce the allocations (e.g., ‘The next item on my list is a bagel, so I’ll put that in my basket. Your next item is a ciabatta, find that one and put it into your basket.’).

As in Experiment 1, the testing phase was preceded by two practice trials. The testing phase was performed on a laptop using PsychoPy3 (Peirce et al., 2019). The probe picture was presented above the three response options. Participants were asked to identify which of the three words was most thematically related to the probe. Participants indicated their responses in the same way as in Experiment 1 (see Experiment 1 ‘Procedure’ section). Self‐reported ratings of response confidence were taken for each trial. Ratings of task enjoyment and focus were not gathered due to the fully randomised design. It was thought that asking participants to self‐report enjoyment and focus after each trial may cause frustration and negatively affect enjoyment or focus.

**Figure 4:**
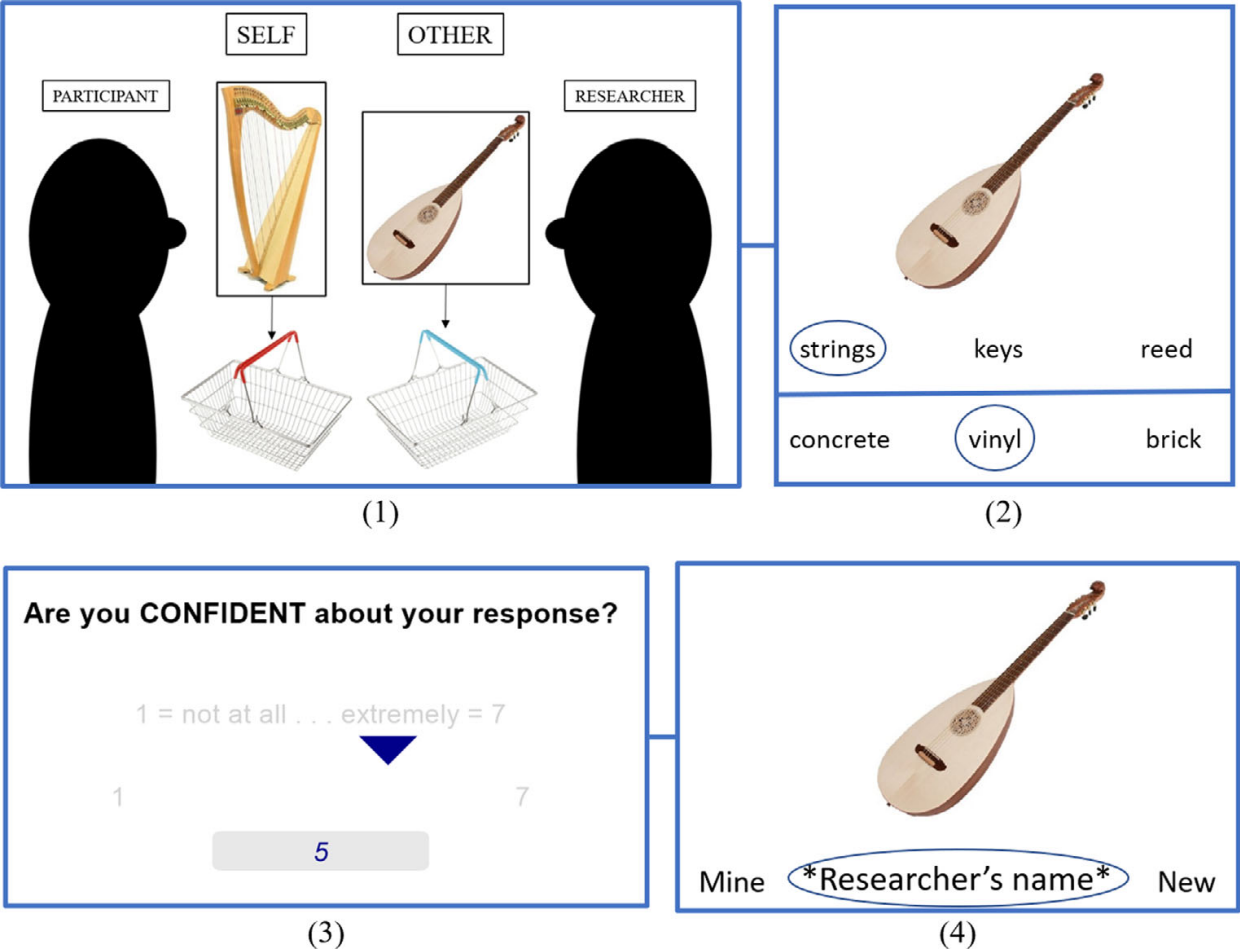
Experiment 2 procedure. (1) One item from each pair of probe pictures was allocated to the participant and the other to the researcher. (2) Participants completed strong and weak associations for both the self‐ and other‐allocated items in each pair. (3) After each trial, participants gave a rating of response confidence. (4) Participants were tested on source memory for 30 pictures.

Finally, participants completed an episodic memory test to test for a self‐reference recognition memory effect, shown previously in SA (Stampacchia et al., 2019). Participants were presented with 30 images, 10 of which had been allocated to them (‘Mine’), 10 which had been allocated to the researcher (‘*researcher’s name*’), and 10 which were not present in the allocation or testing phase (‘New’). Participants indicated which of these three categories they believed each picture belonged to. The same test was administered after both sessions. A summary of the Experiment 2 procedure can be seen in Figure 4.

#### Data analysis

Accuracy (proportion of correct responses) was our key dependent measure. As benefits of self‐reference were only expected for patients on weak association trials, a repeated measures ANOVA was first run for patients only with self‐reference condition (self/other) and association strength (strong/weak) as within‐subjects variables. Accuracy was then entered into an omnibus mixed ANOVA, adding group (patients/controls) as a between‐subjects variable. A mixed ANOVA was conducted for confidence ratings, using the same design as given above. Analysis of participants’ response time can be seen in Table S4. Results of the episodic memory test were analyzed using A’, a non‐parametric measure of recognition memory based on the ratio of correct ‘hits’ to false‐positive responses (Snodgrass & Corwin, 1988). This was calculated for the ‘self’, ‘other’, and ‘new’ conditions. The proportion of correct responses did not vary across sessions for any condition [*p* ≥ .103]. Performance was, therefore, averaged across both sessions. In cases where memory data was only available for one session (*N* = 5), data for this session were entered into the analysis. The effects of group and self‐reference on A’ were assessed using a mixed ANOVA.

### Results

**Figure 5:**
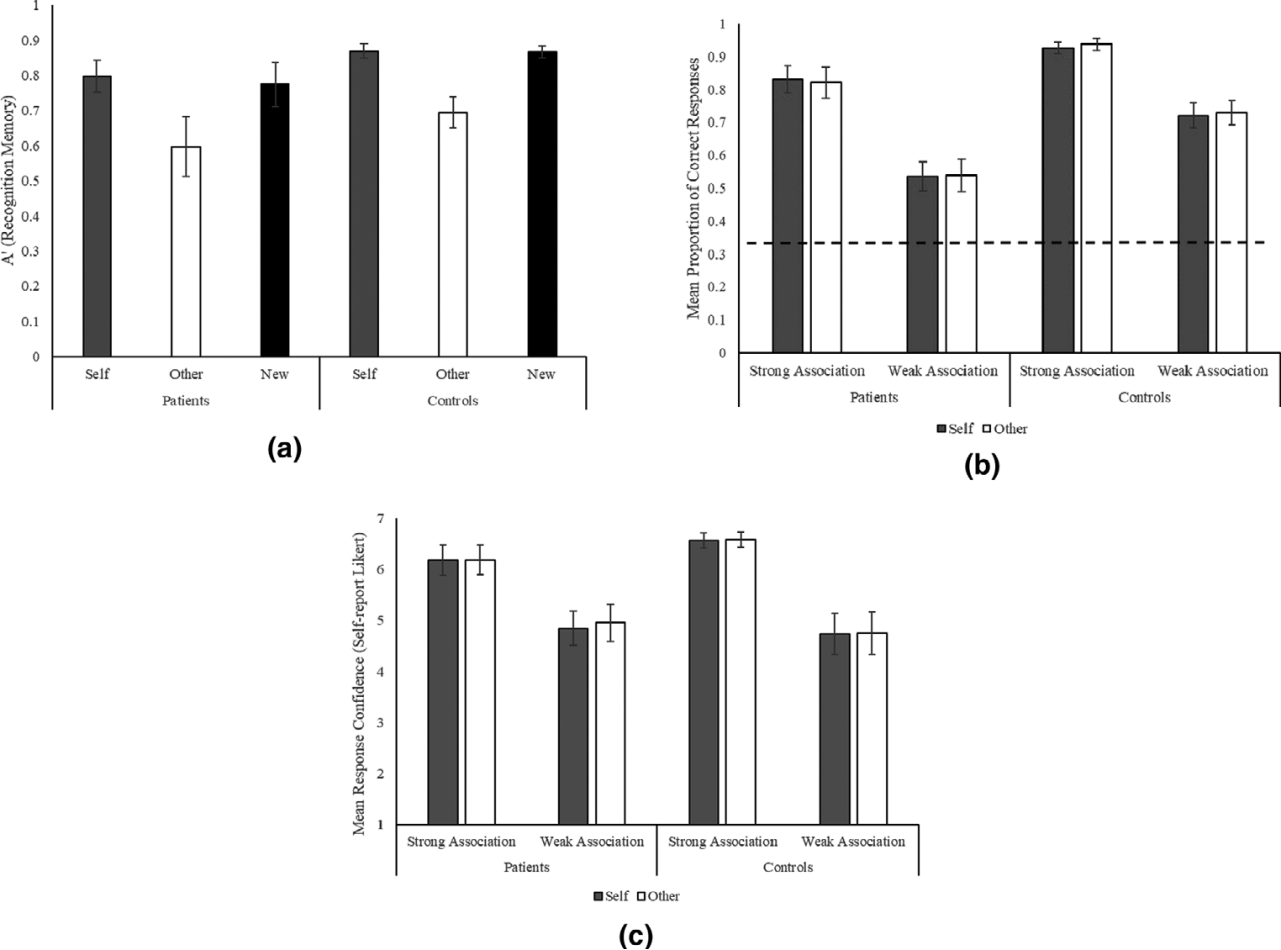
Experiment 2 bar graphs for (a) A’, a non‐parametric signal detection measure of recognition memory based on the proportion of correct hits and false‐positives, (b) mean proportion of correct responses in the semantic task (dotted line = chance), and (c) mean self‐report ratings of response confidence, with standard error of the mean error bars.

Participants’ A’ scores, mean accuracy, and response confidence across self‐reference condition, group, and association strength can be seen in Figure 5. Table S6 provides descriptive statistics for Experiment 2. Results for the recognition memory and omnibus ANOVAs are given in Table 5.

**Table 5:** Omnibus ANOVA results for all Experiment 2 (self‐reference) dependent variables

Dependent variable	Main effect/interaction	Results
A’ (recognition memory)	Group	*F*(1, 19) = 2.1, *p* = .168, $\eta_{p}^{2}$ = .10
	Self‐reference	*F*(1.6, 29.5) = 24.3, *p* < .001, $\eta_{p}^{2}$ = .56^a^
	Self‐reference by group	*F*(1.6, 29.5) = .1, *p* = .861, $\eta_{p}^{2}$ < .01
Accuracy	Group	*F*(1, 20) = 12.7, *p* = .002, $\eta_{p}^{2}$ = .38^a^
	Self‐reference	*F*(1, 20) = .1, *p* = .767, $\eta_{p}^{2}$ < .01
	Self‐reference by group	*F*(1, 20) = .3, *p* = .595, $\eta_{p}^{2}$ = .01
	Strength	*F*(1, 20) = 113.3, *p* < .001, $\eta_{p}^{2}$ = .87^a^
	Strength by group	*F*(1, 20) = 3.7, *p* = .068, $\eta_{p}^{2}$ = .16
	Self‐reference by strength	*F*(1, 20) = .1, *p* = .803, $\eta_{p}^{2}$ < .01
	Self‐reference by strength by group	*F*(1, 20) = .2, *p* = .677, $\eta_{p}^{2}$ < .01
Confidence	Group	*F*(1, 20) = .1, *p* = .776, $\eta_{p}^{2}$ < .01
	Self‐reference	*F*(1, 20) = .3, *p* = .589, $\eta_{p}^{2}$ = .02
	Self‐reference by group	*F*(1, 20) = .1, *p* = .746, $\eta_{p}^{2}$ < .01
	Strength	*F*(1, 20) = 94.9, *p* < .001, $\eta_{p}^{2}$ = .83^a^
	Strength by group	*F*(1, 20) = 2.9, *p* = .102, $\eta_{p}^{2}$ = .01
	Self‐reference by strength	*F*(1, 20) = .2, *p* = .688, $\eta_{p}^{2}$ < .01
	Self‐reference by strength by group	*F*(1, 20) = .2, *p* = .675, $\eta_{p}^{2}$ = .01

^a^ Reflects a significant result at the .05 level.

#### Effects of self‐reference on recognition memory

We report analysis of recognition memory first in order to show the presence of a self‐reference memory effect. Planned comparisons of A’ revealed better recognition memory for self‐allocated than other‐allocated pictures [patients: *t*(9) = 3.0, *p* = .014, controls: *t*(10) = 5.6, *p* < .001], and for new than other‐allocated pictures [patients: *t*(9) = 3.3, *p* = .010, controls: *t*(10) = 4.9, *p* = .001], with no difference between self‐allocated and new pictures [patients: *t* < 1, controls: *t* < 1]. Both groups, therefore, showed the expected self‐reference memory effect and effects of novelty.

#### Effects of self‐reference on semantic retrieval in SA patients

For patients’ semantic judgements, ANOVA revealed a significant main effect of association strength [*F*(1, 9) = 251.1, *p* < .001, $\eta_{p}^{2}$ = .97], with higher accuracy on strong than weak association trials. There was no significant main effect of self‐reference [*F* < 1] or self‐reference by strength interaction [*F* < 1]. Patients’ semantic control composite score positively correlated with overall accuracy [*r*_s_(8) = .90, *p* < .001], reflecting higher accuracy in less‐impaired patients.

#### Omnibus self‐reference ANOVAs

In the omnibus ANOVA including both groups (see Table 5), controls were more accurate than patients. There was a main effect of association strength, reflecting higher accuracy for strong than weak association trials. There were no significant effects or interactions involving self‐reference. Ratings of confidence were higher for strong than weak association trials.

### Experiment 2 summary

Experiment 2 examined the effect of self‐reference on SA patients’ and controls’ ability to make thematic associations. As in Experiment 1, controls were more accurate than the patients, and performance was poorer on weak than strong association trials. Greater response confidence was observed for strong versus weak associations. Despite showing a benefit of self‐reference for recognition memory, self/other‐allocation did not affect the retrieval of thematic associations.

## Discussion

The current study explored the impact of motivation on controlled semantic retrieval in SA patients with multimodal semantic impairment following left frontoparietal stroke. We assessed the impact of performance‐contingent extrinsic reward (Experiment 1) and self‐referentially encoded pictures (Experiment 2) on patients’ and controls’ ability to retrieve strong and weak thematic associations. As expected, SA patients showed lower accuracy overall. Both groups showed lower accuracy for weak associations, thought to reflect higher semantic control demands. Importantly, extrinsic reward improved SA patients’ but not controls’ accuracy. Self‐reference did not impact participants’ semantic performance, despite boosting recognition memory.

SA patients typically show greater semantic impairment for weak associations, when the retrieval of non‐dominant information is required (Thompson et al., 2017). In this study, we did not observe the anticipated interactions between group and association strength in accuracy (or response time, see Table S4), perhaps because the weak association trials were relatively difficult, eliciting frequent errors even in controls, or because our patient sample included mildly impaired individuals. Future research could address these possibilities by observing effects of parametric manipulations of association strength in SA and/or by including more patients with a wider degree of impairment. Experiment 1 demonstrated improvements in participants’ accuracy for weak but not strong associations following high extrinsic reward. SA patients showed an effect of reward while controls did not, suggesting that when sufficient control over semantic retrieval is harder to achieve, benefits of extrinsic reward are maximised. Anticipation of extrinsic reward may increase preparatory cognitive control, supporting the ability to maintain task‐relevant representations and shield against irrelevant information (Goschke & Bolte, 2014). This is consistent with the current finding that high extrinsic reward increased self‐reported task focus. Furthermore, explicit knowledge of task goals has been shown to facilitate semantic judgements (Zhang et al., 2021). Reward may benefit semantic control by augmenting goal maintenance.

Our findings are also consistent with evidence that extrinsic incentives improve performance on domain‐general cognitive control tasks (Capa et al., 2013). Neuroimaging research has shown that introducing extrinsic rewards to cognitive control tasks increases activity across MDN regions (Shashidhara, Mitchell, Erez, & Duncan, 2019), increases functional connectivity between the ventral striatum and MDN (Cubillo, Makwana, & Hare, 2019), and improves decoding accuracy of MVPA classifiers for task‐set information (Etzel, Cole, Zacks, Kay, & Braver, 2016). This reflects enhanced coding of task‐relevant information, in line with suggestions that extrinsic reward improves goal maintenance (Goschke & Bolte, 2014). The interaction between reward and semantic control seen in the current study may also be attributable to modulation of MDN regions, as well as regions specifically recruited during semantic control. Indeed, MDN regions are recruited during semantic tasks with high control demands (Wang, Margulies, Smallwood, & Jefferies, 2020). Future neuroimaging investigations could elucidate the extent to which motivated semantic control is attributable to modulation or recruitment of domain‐general versus semantic control regions. Despite their distinct neurobiological underpinnings (Gao et al., 2021), the current findings suggest that modulatory behavioral effects of reward on semantic control mirror those seen for domain‐general control.

While there is evidence that semantic and domain‐general control are dissociable (Gonzalez Alam, Murphy, Smallwood, & Jefferies, 2018), samples of SA patients can show associations between performance on tests of these functions (Thompson et al., 2018). Semantic and executive control substrates are adjacent, such that damage to one system is frequently accompanied by damage to the other (Souter, Wang, et al., 2021; Wang et al., 2018). Accordingly, the current study revealed a positive correlation between semantic ability and performance on the Brixton Spatial Anticipation Test, a complex nonverbal executive test (Burgess & Shallice, 1997). Patients’ semantic control composite did not correlate with executive measures with verbal requirements, including the difference between parts A and B of the Trail Making Test (Reitan, 1958) or with the nonverbal Raven’s colored progressive matrices (Raven, 1962). While these null results may reflect a lack of statistical power, our results are sufficient to show that associations between semantic and executive performance are not confined to tests with verbal requirements, consistent with evidence that executive performance is independent of verbal demands in aphasia patients with LIFG lesions (Kendrick, Robson, & Meteyard, 2019; see Chapman, Hasan, Schulz, & Martin, 2020 for an alternative view).

The current findings have implications for aphasia rehabilitation. Positive effects of reward are seen in ‘gamification’ strategies to neurorehabilitation (and education more widely), whereby tasks are made more motivating using typical game features, such as rewards and social competition (Landers, 2014). A preliminary investigation demonstrated that gamification may facilitate the rehabilitation of word production following stroke (Romani et al., 2019). The current findings extend this study to show that SA patients can benefit from this strategy, despite deficits of semantic control being accompanied by difficulties in constraining internal representational states in domains beyond semantic cognition, including emotion perception (Souter, Lindquist, et al., 2021) and episodic memory (Stampacchia et al., 2018). These findings merit further investigation of the use of gamified extrinsic incentives in addressing post‐stroke impairments in semantic control. SA patients benefit from external prompts which allude to target concepts, including phonemic cues (Jefferies et al., 2008), context‐relevant sentences (Noonan et al., 2010), and emotional cues (Lanzoni et al., 2019). The current findings demonstrate that prompts which do not provide additional information concerning target concepts, such as abstract extrinsic incentives, can confer similar benefits.

The current manipulation of self‐reference was intended as a proxy for intrinsic motivation, based on evidence of overlapping behavioral effects of self‐reference and reward processing (Sui & Humphreys, 2015a) and overlapping neural substrates underlying self‐reference and intrinsic motivation (Tamir & Mitchell, 2012). We found expected effects of self‐reference on recognition memory, suggesting that we successfully evoked self‐referential encoding, consistent with prior evidence from SA (Stampacchia et al., 2019). Self‐reference was not found to modulate semantic retrieval. This null result does not preclude the role of intrinsic motivation in semantic performance; the manipulation in the current study may have been insufficient. In future studies, further tailoring may be required to elicit stronger intrinsic motivators. As intrinsic motivation reflects inherent interest or enjoyment (Mori et al., 2018), it may be beneficial to include stimuli which are specifically of interest to, or belong to, the participant.

### Limitations

The current study is limited insofar as we did not measure several constructs related to reward processing. Affective abnormalities, including apathy (Fishman et al., 2018) and hypo/hyperarousal (Heilman, Schwartz, & Watson, 1978; Laures, Odell, & Coe, 2003), are common following stroke, and could interfere with reward sensitivity. This has been demonstrated in relation to apathy, following damage to subcortical reward processing regions (Rochat et al., 2013). The current study cannot account for these effects. It is worth noting, however, that in the current sample, subcortical and medial regions were relatively intact (see ‘Lesion analyses’ section). Future investigations into reward processing in post‐stroke aphasia may benefit from measuring apathy, reward sensitivity, and physiological arousal to better account for effects of these constructs.

### Conclusion

The current study demonstrates that extrinsic reward can improve SA patients’ ability to make thematic associations. As with domain‐general cognitive control, extrinsic reward may bolster semantic retrieval through increased proactive control. These findings have practical implications for the rehabilitation of post‐stroke semantic impairment; language therapy activities for SA patients could be facilitated using a gamification‐based approach incorporating external rewards. Effects of self‐reference on semantic performance were not observed.

## Funding information

This project was funded by Wellcome through the Centre for Future Health at the University of York and by a project grant from the Stroke Association (TSA/12/02). EJ was funded by an ERC Consolidator grant (FLEXSEM – 771863).

## Conflict of interest

The authors have no competing interests to disclose.

## Author contribution

**Nicholas Edward Souter:** Conceptualization (equal); Data curation (equal); Formal analysis (equal); Investigation (equal); Methodology (equal); Project administration (equal); Software (equal); Validation (equal); Writing – original draft (equal); Writing – review & editing (equal). **Sara Stampacchia:** Conceptualization (equal); Data curation (equal); Investigation (equal); Methodology (equal); Project administration (equal); Software (equal); Validation (equal); Writing – review & editing (equal). **Glyn Hallam:** Conceptualization (equal); Investigation (equal); Writing – review & editing (equal). **Hannah Thompson:** Conceptualization (equal); Writing – review & editing (equal). **Jonathan Smallwood:** Conceptualization (equal); Writing – review & editing (equal). **Elizabeth Jefferies:** Conceptualization (equal); Funding acquisition (equal); Methodology (equal); Supervision (equal); Writing – review & editing (equal).

## Supporting information

Table S1 Stimulus properties for Experiment 1.
Table S2 Stimulus properties for Experiment 2.
Table S3 ANOVAs for Experiment 1 and Experiment 2 psycholinguistic factors.
Table S4 Analysis and interpretation of response time (seconds) in Experiment 1 and Experiment 2.
Table S5 Descriptive statistics for Experiment 1.
Table S6 Descriptive statistics for Experiment 2.

## Data Availability

Data sharing *via* a public repository is not possible for the current study due to insufficient consent. Researchers who wish to access the data should contact the Research Ethics Committee of the York Neuroimaging Centre or the corresponding author.

## Footnotes

^1^
The assumption of normality was not always met, but non‐parametric tests elicited the same outcomes. Weak associations: *Z* = −2.9, corrected *p* = .008; strong associations: *Z* = −.2, corrected *p* > 1.

^2^
[Patients: *Z* = −2.1, corrected *p* = .070, controls: *Z* = −.4, corrected *p* > 1].

^3^
[Weak association: *Z* = −2.4, corrected *p* = .034, strong association: *Z* = −.7, corrected *p* = .976].
